# Breast Milk: A Potential Route of Tick-Borne Encephalitis Virus Transmission from Mother to Infant

**DOI:** 10.7759/cureus.41590

**Published:** 2023-07-09

**Authors:** Rohit Dabas, Nandita Sharma, Avinash B Taksande, Roshan Prasad, Pratiksha K Munjewar, Mayur B Wanjari

**Affiliations:** 1 Internal Medicine, Jawaharlal Nehru Medical College, Datta Meghe Institute of Higher Education and Research, Wardha, IND; 2 Physiology, Jawaharlal Nehru Medical College, Datta Meghe Institute of Higher Education and Research, Wardha, IND; 3 Medical Surgical Nursing, Smt. Radhikabai Meghe Memorial College of Nursing, Datta Meghe Institute of Higher Education and Research, Wardha, IND; 4 Research and Development, Jawaharlal Nehru Medical College, Datta Meghe Institute of Higher Education and Research, Wardha, IND

**Keywords:** prevention, clinical implications, infants, transmission, breast milk, tick-borne encephalitis virus (tbev)

## Abstract

Tick-borne encephalitis virus (TBEV) is a global public health concern, and understanding its transmission routes is crucial for effective prevention and control. While tick bites are the primary mode of TBEV transmission, emerging evidence suggests the potential for TBEV transmission through breast milk from infected mothers to their infants. This review article provides an overview of the current knowledge regarding TBEV transmission through breast milk and its clinical implications. It explores the presence and persistence of TBEV in breast milk, potential mechanisms of transmission, and the role of immune factors in facilitating or inhibiting viral transmission. The clinical outcomes and complications in infants infected with TBEV through breast milk are discussed, along with the epidemiological patterns and geographical considerations of this transmission mode. Preventive and management strategies are also addressed, including public health measures, risk assessment, and potential interventions. Future research directions are highlighted, emphasizing the need for further epidemiological studies, investigations into viral load dynamics, immune responses, and the development of preventive measures targeting TBEV transmission through breast milk. By expanding our knowledge in these areas, we can improve strategies to reduce the risk of TBEV transmission from mothers to infants and protect vulnerable populations.

## Introduction and background

Tick-borne encephalitis (TBE) is a viral infection caused by the tick-borne encephalitis virus (TBEV). TBEV is primarily transmitted to humans through the bite of infected ticks, with ticks belonging to the *Ixodes* genus being the main vectors [1]. TBE poses a significant public health concern, especially in regions where ticks are prevalent, such as parts of Europe, Asia, and North America. The clinical manifestations of TBEV infection can range from mild flu-like symptoms to severe neurological complications, including meningitis, encephalitis, and long-term cognitive impairments [1,2].

While ticks are the primary vectors for TBEV transmission, emerging evidence suggests that other transmission routes, such as breast milk, may also play a role in spreading the virus. This raises concerns about the potential risks associated with TBEV infection in infants through breastfeeding. Breast milk is widely recognized as the optimal method of infant nutrition, providing numerous health benefits to both the mother and child. However, if breast milk becomes a potential route of TBEV transmission, it introduces a new dimension of concern for breastfeeding mothers in regions where TBEV is endemic [3].

Understanding the transmission routes and potential risks associated with TBEV infection in infants is crucial for public health interventions and strategies. It is important to assess the possibility of TBEV transmission through breast milk and evaluate the implications for breastfeeding recommendations in regions affected by TBE. This knowledge can help inform healthcare professionals, policymakers, and lactating mothers about the potential risks and guide decision-making regarding breastfeeding practices in TBEV-endemic areas. By comprehensively examining the evidence and understanding the dynamics of TBEV transmission, effective preventive measures can be developed to ensure the safety and well-being of mothers and infants in regions affected by TBE [4].

This review explores the hypothesis that breast milk could be a potential route for TBEV transmission from mothers to infants. By examining the existing literature on TBEV, breastfeeding, and viral transmission through breast milk, this review aims to assess the evidence and shed light on the potential risks and implications of TBEV transmission through breastfeeding.

## Review

Methodology

**Figure 1:**
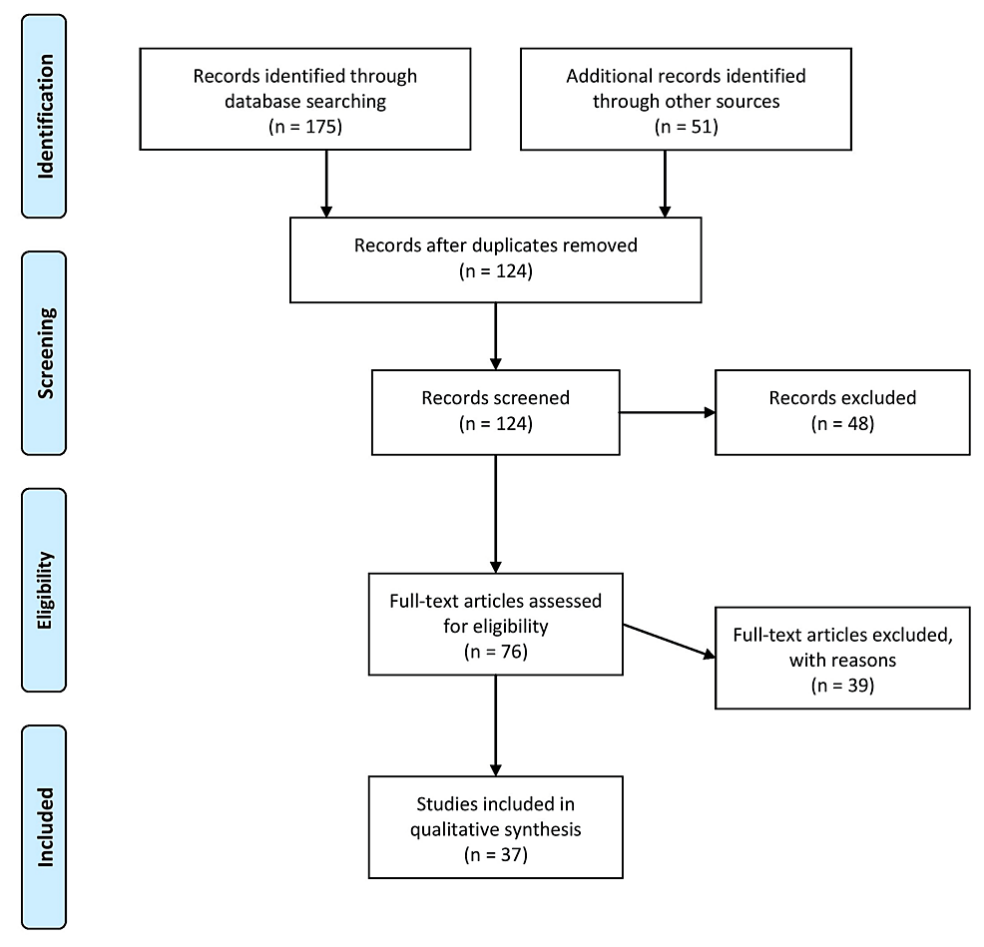
Selection process of articles used in this study. Adopted from the Preferred Reporting Items for Systematic Reviews and Meta-Analyses (PRISMA)

This review systematically gathered relevant literature on TBEV transmission through breast milk. A comprehensive search strategy was developed using electronic databases, including PubMed, Scopus, and Web of Science. The search terms included variations of “TBEV,” “breastfeeding,” “breast milk,” “transmission,” “infants,” and “related keywords.” The search was conducted without language or time restrictions up until the knowledge cut-off date of September 2021. The selection criteria for the included studies were determined based on relevance to the topic and the quality of the evidence. Studies eligible for inclusion investigated TBEV transmission through breast milk through primary data collection or systematic reviews. Studies focusing on other modes of TBEV transmission or unrelated topics were excluded. The inclusion criteria encompassed experimental and observational studies, including case reports, cohort studies, cross-sectional studies, and clinical trials. Animal studies were also considered if they provided insights into the mechanisms or dynamics of TBEV transmission through breast milk. During the study selection process, titles and abstracts were screened, followed by a full-text review of potentially relevant articles. Any discrepancies in study selection were resolved through consensus among the reviewers. The final set of included studies provides a comprehensive overview of the available evidence on TBEV transmission through breast milk, encompassing clinical and experimental research. The findings from these studies were synthesized and analyzed to draw meaningful conclusions and identify areas requiring further investigation. Figure 1 describes the selection process of articles used in our study.

TBEV

A Brief Introduction to TBEV, Its Classification, and Geographic Distribution

TBEV is a member of the Flaviviridae family, including other medically important viruses, such as dengue, Zika, and West Nile. TBEV is further classified into three subtypes: European (TBEV-Eu), Siberian (TBEV-Sib), and Far Eastern (TBEV-FE). These subtypes vary in their geographic distribution and disease severity [5,6]. TBEV has a wide geographic distribution, primarily in parts of Europe and Asia. Endemic regions include central and eastern European countries (e.g., Austria, Czech Republic, Germany, and Russia), Scandinavia, the Baltic states, China, Mongolia, and parts of Japan. The incidence and prevalence of TBEV infections vary across these regions, with some areas reporting sporadic cases while others experiencing periodic outbreaks [7].

Lifecycle and Transmission of TBEV, Emphasizing Tick Vectors and Their Role

The lifecycle of TBEV involves ticks and vertebrate hosts, typically small mammals, such as rodents and insectivores. Ticks serve as both vectors and reservoirs for TBEV. The primary vector species responsible for TBEV transmission are *Ixodes ricinus* in Europe and *Ixodes persulcatus* in Asia [8,9]. Ticks become infected with TBEV when they feed on infected animals. The virus then replicates within the tick, infecting various tissues, including salivary glands. Infected ticks can transmit the virus to humans and other animals during subsequent feeding [10].

Transmission to humans usually occurs through the bite of an infected tick. TBEV is mainly transmitted during feeding when the tick regurgitates virus-containing saliva into the host's skin. It is important to note that not all tick bites result in TBEV transmission, as the ticks themselves must be infected with the virus [11].

Clinical Presentation and Potential Complications of TBE in Infants

The clinical presentation of TBE can vary from mild to severe, with symptoms typically appearing 7-14 days after an infected tick bite. In infants, TBE may present differences compared to older children and adults. Infants are generally more susceptible to severe forms of the disease [12]. Initial symptoms of TBE in infants can be non-specific and resemble common viral illnesses, including fever, fatigue, headache, and muscle aches. As the disease progresses, neurological symptoms may emerge, such as meningitis (inflammation of the membranes surrounding the brain and spinal cord) or encephalitis (inflammation of the brain) [3,4,10].

Severe cases of TBE in infants can lead to neurological complications, including seizures, cognitive impairments, and motor abnormalities. Long-term consequences, such as learning disabilities and behavioral problems, have been reported in some cases. It is important to note that not all infants infected with TBEV will develop severe symptoms, and the severity of the disease can vary [13]. The clinical manifestations and potential complications of TBE in infants highlight the importance of understanding transmission routes and potential risks, such as TBEV transmission through breast milk. Further investigation into this potential transmission route is crucial to inform preventive measures and ensure the safety of breastfeeding practices in TBE-endemic areas [6,9].

Breast milk as a transmission route

Overview of Breastfeeding and Its Benefits for Infant Health

Breastfeeding is widely recognized as the optimal method of infant nutrition and is associated with numerous health benefits for both the infant and mother. Breast milk provides complete nutrition, containing nutrients, growth factors, hormones, enzymes, and immune-protective components that support the infant's growth and development [14,15].

Breast milk offers several advantages for infant health. First, it provides optimal nutrition as it contains a balanced combination of essential nutrients, proteins, fats, carbohydrates, vitamins, and minerals specifically tailored to meet the nutritional needs of infants, supporting their healthy growth and development. Second, breast milk provides immune protection through its rich content of immune factors, such as antibodies, leukocytes, and antimicrobial components. These components help to strengthen the infant's immune system and protect against various infections, reducing the risk of respiratory and gastrointestinal infections. Third, breastfeeding has been associated with a reduced risk of allergies, asthma, obesity, and certain chronic diseases later in life, potentially due to the immune-boosting properties of breast milk. Finally, breast milk contains factors that promote cognitive development and may contribute to better cognitive outcomes in infants. The unique composition of breast milk provides numerous benefits that support the overall health and well-being of infants [16].

The Concept of Viral Transmission Through Breast Milk

Breast milk can be a potential viral transmission route from mothers to infants. Viruses can be present in breast milk either due to a localized infection in the mammary glands or due to viremia (presence of virus in the bloodstream) in the mother. Viruses in breast milk raise concerns about the potential risk of transmission and the impact on infant health [17]. Not all viruses can be transmitted through breast milk, and the transmission risk varies depending on the specific virus, viral load, and other factors. Breast milk viruses can be transmitted by ingesting viral particles or through infected cells present in breast milk [18].

Evidence of Viral Transmission Through Breast Milk in Other Diseases

Several viruses have been documented to be transmitted through breast milk in different disease contexts. Examples include human immunodeficiency virus (HIV), human T-cell lymphotropic virus type 1 (HTLV-1), cytomegalovirus (CMV), herpes simplex virus (HSV), and hepatitis C virus (HCV) [19]. Studies have shown that these viruses can be present in breast milk and infect the infant during breastfeeding. However, the transmission rates and risk factors vary for each virus, and preventive strategies, such as antiretroviral therapy for HIV-positive mothers, can significantly reduce transmission rates [20,21].

The Rationale for Investigating TBEV Transmission Through Breast Milk

Given the potential for viral transmission through breast milk, it is important to investigate whether TBEV can be transmitted through this route. Understanding the potential for TBEV transmission through breast milk is essential for several reasons:

Public health implications: If TBEV can be transmitted through breast milk, it has important implications for public health strategies and breastfeeding recommendations in TBE-endemic areas. Guidance can be developed to minimize the risk of transmission while still promoting the numerous benefits of breastfeeding [8,15,21]. 

Infant health outcomes: Investigating TBEV transmission through breast milk is crucial to determine the potential impact on infant health. Understanding the clinical consequences and long-term outcomes of TBEV transmission through breastfeeding can guide appropriate interventions and management strategies [15,16].

Prevention and interventions: Identifying the mechanisms and risk factors for TBEV transmission through breast milk can help develop preventive measures and interventions to reduce the transmission risk. This knowledge can inform public health efforts and contribute to controlling and preventing TBEV infections [13,17]. By examining the existing evidence and exploring the potential for TBEV transmission through breast milk, this review aims to provide valuable insights into the risk factors, mechanisms, and clinical implications of TBEV transmission in the context of breastfeeding.

Presence and persistence of TBEV in breast milk

Studies Assessing the Presence of TBEV in Breast Milk Samples

Several studies have investigated the presence of TBEV in breast milk samples to determine if breast milk can be a potential transmission route from mothers to infants. These studies have employed various methodologies to detect and analyze the presence of TBEV in breast milk [2,4,6,8,10,21]. Studies have collected breast milk samples from TBEV-infected mothers during different stages of infection and have used techniques, such as RT-PCR and nucleic acid amplification tests, to detect viral RNA. Other studies have employed virus isolation techniques to culture TBEV from breast milk samples [2,4,6,8,10,21].

Detection Methods Used to Identify TBEV in Breast Milk

The detection of TBEV in breast milk samples typically involves molecular techniques, such as RT-PCR or real-time PCR, which allow for the amplification and detection of specific viral RNA sequences. These methods rely on TBEV genetic material (RNA) in the breast milk sample [4,15]. In addition to molecular techniques, immunological methods, such as enzyme-linked immunosorbent assays (ELISAs) or immunofluorescence assays, can detect TBEV antigens or antibodies in breast milk. These methods can provide indirect evidence of the presence of TBEV or previous exposure to the virus [4,5].

Factors Influencing the Presence and Persistence of TBEV in Breast Milk

Several factors can influence the presence and persistence of TBEV in breast milk:

Maternal viremia: The presence of TBEV in breast milk can be influenced by the mother's level of viremia (virus in the bloodstream). High viremia levels may increase the likelihood of TBEV transmission to breast milk [9,18].

Timing of infection: The timing of TBEV infection during breastfeeding can affect the presence of the virus in breast milk. Studies have reported higher viral loads in breast milk samples collected during the acute phase of infection compared to convalescent or post-convalescent stages [11,12,14,19].

Mammary gland involvement: TBEV may gain access to breast milk through the mammary glands. In cases where the virus is present in the mammary gland tissue, the likelihood of viral presence in breast milk increases [8].

Individual variations: There may be individual variations in viral shedding and persistence in breast milk among TBEV-infected mothers. The mother's immune response, viral replication dynamics, and genetic factors may contribute to these variations [17].

Viral Load and Its Correlation With TBEV Transmission Potential

The viral load, or the amount of TBEV present in breast milk, may be associated with the potential for transmission to the infant. Higher viral loads in breast milk may indicate a higher risk of transmission. However, the correlation between viral load and transmission potential is not yet well established. Studies have reported a wide range of viral loads in breast milk samples, with some showing low or undetectable viral loads. Further research is needed to determine the threshold viral load necessary for transmission and to understand the relationship between viral load and the likelihood of transmission [21]. Investigating the factors influencing the presence and persistence of TBEV in breast milk, as well as the correlation between viral load and transmission potential, can provide valuable insights into the potential risk of TBEV transmission through breastfeeding and guide the development of preventive strategies to minimize transmission risks [4,6,8,15,16,21].

Mechanisms of TBEV transmission through breast milk

Possible Mechanisms by Which TBEV May Cross From Maternal Blood to Breast Milk

The exact mechanisms by which TBEV crosses from maternal blood to breast milk are not fully understood. However, several potential pathways have been proposed:

Hematogenous dissemination: TBEV can enter the bloodstream through the bite of an infected tick and subsequently disseminate to various organs and tissues, including the mammary glands. This suggests that TBEV may have the ability to cross from the maternal blood into the mammary gland tissue and subsequently be secreted into breast milk. The exact mechanisms by which TBEV can traverse from the blood to the mammary glands are not fully understood and require further investigation [22].

Transcytosis: Transcytosis is a process by which molecules are transported across cells. It is hypothesized that TBEV may utilize transcytosis to cross the mammary epithelial cells, which form a barrier between the bloodstream and the milk-producing alveoli within the mammary glands. The virus could pass through the mammary epithelial cells and enter breast milk through transcytosis. This mechanism requires exploration to understand better the specific cellular processes involved and the factors that may facilitate or hinder TBEV's transcytosis across the mammary epithelium [23].

Mammary gland infection: Another potential mechanism for TBEV transmission through breast milk is the direct infection of the mammary gland’s glandular tissue. It is conceivable that TBEV could infect the mammary epithelial cells within the mammary glands, leading to viral replication within these cells. As a result, the virus could be released into breast milk due to the infected mammary gland tissue. Further investigation is needed to ascertain the susceptibility of mammary epithelial cells to TBEV infection and the subsequent release of the virus into breast milk [24]. The specific mechanisms involved in TBEV transmission to breast milk are an area of ongoing research and require further investigation to gain a comprehensive understanding.

Role of Immune Factors in Breast Milk in Facilitating or Inhibiting Viral Transmission

Breast milk contains various immune factors that can play a role in facilitating or inhibiting viral transmission. These immune factors include antibodies, immune cells, cytokines, and other soluble factors.

Antibodies: Breast milk contains many antibodies, primarily secretory immunoglobulin A (IgA). These antibodies can specifically target and neutralize viruses, preventing their entry into cells and reducing the risk of viral transmission. IgA antibodies provide localized protection within the mucosal surfaces of the infant's digestive and respiratory tracts, which are common entry points for viral infections [25,26].

Immune cells: Breast milk is abundant in antibodies and contains various immune cells, including lymphocytes and macrophages. These immune cells can recognize and eliminate pathogens, including viruses, in breast milk. By clearing viral particles and infected cells, breast milk's immune cells help minimize the potential for viral transmission to the infant [27].

Cytokines and other immune factors: Breast milk also contains a diverse range of cytokines and other immune factors contributing to its protective properties. These immune factors can modulate the immune response in the infant, promoting antiviral defense and reducing the susceptibility to viral infections. In addition, they can enhance the overall immune maturation and development of the infant's immune system [28]. The presence of these immune factors in breast milk suggests a potential protective role against viral transmission. However, the effectiveness of these immune factors in preventing TBEV transmission specifically requires further investigation.

Interactions Between TBEV and Mammary Epithelial Cells

TBEV may interact with mammary epithelial cells during viral entry into breast milk. The exact mechanisms and interactions involved in this process are not fully understood but may include the following:

Receptor-mediated entry: TBEV may utilize specific receptors present on the surface of mammary epithelial cells to facilitate viral entry. The interaction between TBEV and these receptors is hypothesized to initiate cellular events that allow the virus to enter the mammary epithelial cells. However, the specific receptors involved in the TBEV entry into mammary epithelial cells have not been definitively identified yet. Further research is needed to elucidate the receptor-virus interactions and understand this process' mechanisms [29].

Intracellular trafficking: Once TBEV gains entry into mammary epithelial cells, it may undergo intracellular trafficking, utilizing cellular pathways for the transport, replication, and assembly of new virus particles. The virus may exploit various cellular machinery and compartments within the mammary epithelial cells to establish replication sites and replicate its genetic material. The exact intracellular trafficking pathways and the host factors facilitating TBEV replication and assembly in mammary epithelial cells require further investigation [30].

Viral release: Following replication and assembly, TBEV has the potential to be released from mammary epithelial cells into breast milk. The exact mechanisms of viral release are not fully understood but may involve processes, such as exocytosis or other cellular mechanisms. Once released into breast milk, TBEV can potentially be ingested by the infant during breastfeeding, leading to virus transmission. The dynamics of viral release from mammary epithelial cells and the factors influencing the efficiency of this process warrant further exploration [2,20,29]. Understanding the interactions between TBEV and mammary epithelial cells is important for elucidating the mechanisms of TBEV transmission through breast milk. Further research is needed to investigate these interactions and the specific molecular mechanisms involved.

Clinical implications and epidemiological considerations

Case Reports or Studies Documenting Potential TBEV Transmission Through Breast Milk

Several case reports and studies have reported the potential transmission of TBEV through breast milk. These reports have documented instances where infants have acquired TBEV infection through breastfeeding. However, it is important to note that these cases are relatively rare, and the overall risk of TBEV transmission through breast milk appears low [9,10]. These case reports and studies have described TBEV infection in infants breastfed by mothers with confirmed TBEV infection. TBEV RNA, or the isolation of live viruses from breast milk samples, has been reported in some cases. These findings suggest the possibility of TBEV transmission through breast milk [1,5,9,24,25,27].

Clinical Outcomes and Complications in Infants Infected With TBEV via Breast Milk

The clinical outcomes and complications in infants infected with TBEV via breast milk can vary. Some infants may remain asymptomatic or develop mild symptoms, while others may experience more severe manifestations of TBE [31]. Infants infected with TBEV via breast milk can present with symptoms, such as fever, irritability, poor feeding, lethargy, and neurological manifestations, including seizures and encephalitis. In severe cases, TBEV infection can lead to long-term neurological sequelae or even death [32].

The severity of clinical outcomes in infants may depend on various factors, including the viral load in breast milk, the timing of infection, the immune response of the infant, and other individual factors. Further research is needed to better understand the clinical spectrum and outcomes of TBEV transmission through breast milk [33].

Epidemiological Patterns and Geographical Considerations of TBEV Transmission Through Breast Milk

The epidemiological patterns of TBEV transmission through breast milk vary in different regions. Geographical considerations play a significant role due to variations in TBEV prevalence and endemicity [16]. TBEV is endemic in certain regions of Europe and Asia, with the highest incidence reported in central and eastern European countries and parts of Russia and China. In these endemic areas, the risk of TBEV infection is generally higher, and the potential for transmission through breast milk may be more significant. Geographical factors, such as the prevalence of TBEV-infected ticks in the environment, incidence of TBEV infection in the population, and overall epidemiological context of TBEV transmission, can influence the likelihood of TBEV transmission through breast milk [34].

Healthcare providers and public health authorities need to consider the local epidemiological patterns and prevalence of TBEV when assessing the risk of transmission through breast milk. This information can help inform preventive strategies, vaccination recommendations, and public health interventions to minimize the risk of TBEV transmission in breastfeeding infants. Further epidemiological studies are needed to understand better the geographical distribution and risk factors associated with TBEV transmission through breast milk and to guide targeted prevention and control measures [5,24].

Prevention and management strategies

Importance of Public Health Measures to Prevent TBEV Transmission

Public health measures are crucial in preventing TBEV transmission, including the potential transmission through breastfeeding. These measures are essential for reducing the overall burden of TBEV infection in the community and protecting vulnerable populations, such as infants. Public health measures for TBEV prevention typically include the following:

Tick bite prevention: Educating the public about tick bite prevention strategies is crucial in reducing the risk of TBEV infection in adults and infants. This includes raising awareness about the importance of wearing protective clothing, such as long sleeves and pants, using insect repellents containing DEET (N,N-diethyl-m-toluamide) or permethrin, and avoiding tick-infested areas, such as tall grasses and wooded areas. Implementing these preventive measures can significantly decrease the likelihood of tick bites and subsequent TBEV transmission [2,5].

Vaccination: Vaccination is the most effective preventive measure against TBEV infection. In endemic regions where TBEV is prevalent, vaccination campaigns should be implemented targeting at-risk populations, including lactating mothers. Vaccinating lactating mothers can confer passive immunity to their infants through breast milk, protecting against TBEV transmission. It is important to ensure that the safety and efficacy of TBEV vaccines in lactating mothers are well established through clinical trials and monitoring [35,36].

Surveillance and early detection: Establishing robust surveillance systems to monitor TBEV activity is essential for early detection and prompt public health interventions. Surveillance programs should include monitoring tick populations, TBEV prevalence in ticks, and human TBEV cases. The timely detection of TBEV-infected individuals, including lactating mothers, enables appropriate management, including medical care and counseling on breastfeeding options. In addition, surveillance data can inform public health interventions, such as targeted tick control measures and awareness campaigns in high-risk areas [37].

Strategies for Minimizing the Risk of TBEV Transmission Through Breastfeeding

Minimizing the risk of TBEV transmission through breastfeeding requires a comprehensive approach that considers individual and public health measures. Some strategies to minimize the risk include the following:

Education and awareness: Providing comprehensive education and awareness programs to lactating mothers about TBEV transmission and the potential risks associated with breastfeeding during active TBEV infection is crucial. Mothers should be informed about the symptoms of TBEV infection and advised to seek medical advice promptly if they develop any relevant symptoms. This education empowers mothers to make informed decisions regarding breastfeeding, considering the potential risks and benefits [18,24,25].

Screening and testing: Implementing screening measures for TBEV infection in lactating mothers, particularly in endemic regions, can help identify individuals at risk of transmitting the virus through breast milk. Diagnostic testing, such as reverse transcription-polymerase chain reaction (RT-PCR), can be used to detect the presence of TBEV RNA in breast milk samples. Screening and testing can aid in identifying and appropriately managing TBEV-infected individuals [9,18].

Individual risk assessment: Healthcare providers should conduct individual risk assessments for lactating mothers suspected or confirmed to have TBEV infection. Factors, such as the stage of infection, viral load in breast milk, clinical presentation, and prevalence of TBEV in the local population, should be considered. The risk assessment helps determine the appropriateness of breastfeeding and guides decisions regarding the best course of action for each individual [13,17].

Temporary cessation or alternative feeding options: In cases where the risk of TBEV transmission through breastfeeding is deemed significant based on the individual risk assessment, temporary cessation of breastfeeding or the provision of safe alternative feeding options can be considered. This decision should be made on a case-by-case basis, considering factors, such as the severity of maternal infection, availability of expressed breast milk, and feasibility of formula feeding. Safe alternative feeding options, such as expressed breast milk from uninfected donors or commercial infant formula, can be considered to ensure adequate nutrition for the infant while minimizing the risk of TBEV transmission [13,31].

Immunization and Vaccine Considerations for Lactating Mothers

Immunization against TBEV is a crucial preventive strategy, and the vaccination status of lactating mothers should be considered. TBEV vaccines are generally safe and effective; in some endemic regions, immunization of lactating mothers may be recommended [37]. Current evidence suggests that TBEV vaccines do not pose a significant risk to lactating infants. Inactivated TBEV vaccines are not live vaccines and are unlikely to replicate or cause infection in the infant through breast milk. However, the specific recommendations regarding TBEV vaccination for lactating mothers may vary between countries and should be based on available guidelines and expert advice [3].

Healthcare providers should consider the benefits and potential risks of TBEV vaccination in lactating mothers, considering the local epidemiological context, individual's risk of TBEV infection, and potential benefits of breastfeeding for the infant's health. Shared decision-making between the mother and healthcare provider is essential in determining the most appropriate action. It is important to regularly review and update vaccination guidelines and recommendations for lactating mothers based on emerging evidence and the evolving epidemiological situation [1,13,18].

Future research directions

Research Gaps and Areas Requiring Further Investigation

Despite existing knowledge on TBEV transmission through breast milk, several research gaps must be addressed to enhance our understanding of this transmission mode. Areas requiring further investigation include the following:

Epidemiological studies: Conducting large-scale epidemiological studies is crucial to determine the incidence and prevalence of TBEV transmission through breast milk in different geographic regions. These studies can help assess the overall risk and burden of transmission and identify specific risk factors associated with TBEV transmission through breastfeeding. By collecting comprehensive data on TBEV-infected lactating mothers and their infants, researchers can better understand this transmission mode's epidemiological patterns and geographical variations.

Mechanisms of transmission: Elucidating the precise mechanisms by which TBEV crosses from maternal blood to breast milk is essential for understanding TBEV transmission through breastfeeding. Investigating the viral and host factors involved in viral entry, replication, and release in mammary epithelial cells can provide insights into the specific pathways and interactions underlying TBEV transmission through breast milk. By studying the molecular mechanisms involved, researchers can identify potential targets for interventions to block or inhibit viral transmission.

Viral load dynamics: Understanding the dynamics of TBEV viral load in breast milk over time and its correlation with the risk of transmission is critical for assessing the potential infectivity of breast milk. Longitudinal studies that track the viral load in breast milk samples from TBEV-infected mothers can provide valuable information on the kinetics of TBEV shedding and the factors that influence viral load dynamics. This knowledge can help determine the appropriate duration and precautions for lactating mothers to minimize the risk of transmission.

Immune response: Investigating the role of the maternal immune response in preventing or facilitating TBEV transmission through breast milk is essential for understanding the factors that influence viral transmission. Studying the specific immune factors and mechanisms involved in the antiviral defense within breast milk can help identify potential targets for interventions. By enhancing our understanding of the immune response, researchers can explore strategies to boost protective immune factors in breast milk or develop interventions that modulate the immune environment to reduce the risk of viral transmission.

Clinical outcomes: Conducting larger-scale studies to further characterize the clinical outcomes and long-term sequelae in infants infected with TBEV through breast milk is crucial for understanding the impact of TBEV transmission on infant health. These studies can provide a more comprehensive understanding of the spectrum of clinical manifestations, potential complications, and long-term effects of TBEV infection acquired through breastfeeding. By evaluating the clinical outcomes in a larger cohort of infected infants, healthcare providers can improve the management and care of affected infants and provide appropriate counseling and support to affected families.

Prospects for Developing Preventive Measures Targeting TBEV Transmission Through Breast Milk

Developing preventive measures targeting TBEV transmission through breast milk is an important area for future research. Prospects for such measures include the following:

Vaccine development: A promising avenue is investigating the potential for developing vaccines specifically targeting TBEV transmission through breast milk. This could involve the development of vaccines that can induce local immune responses within the mammary gland tissue. These vaccines aim to prevent viral entry and replication by targeting the mammary gland, ultimately reducing the risk of TBEV transmission through breast milk. Further research is needed to explore the feasibility and effectiveness of such vaccines, including preclinical and clinical studies [17,1,33].

Antiviral therapies: Another potential intervention to consider is exploring the efficacy of antiviral therapies in reducing viral load and inhibiting TBEV replication in breast milk. Identifying effective antiviral agents that can be safely administered to lactating mothers may provide an additional tool for reducing the risk of TBEV transmission. Research should focus on assessing the safety, efficacy, and optimal dosage of antiviral therapies, specifically in lactating women, and their impact on TBEV transmission through breast milk [32,33].

Maternal immunization: Further evaluating the safety and efficacy of TBEV vaccination in lactating mothers can have dual benefits of preventing both maternal and infant TBEV infections. Investigating the immune response elicited by vaccination in lactating mothers is crucial, including the transfer of vaccine-induced antibodies to breast milk. This research can provide valuable insights into the potential of maternal immunization as a preventive strategy to reduce the risk of TBEV transmission through breast milk. Large-scale studies and clinical trials are needed to assess the safety, immunogenicity, and long-term protection conferred by TBEV vaccines in lactating mothers and their infants [29,36].

Potential Interventions to Reduce the Risk of TBEV Transmission From Mothers to Infants

To reduce the risk of TBEV transmission from mothers to infants, potential interventions can be explored, including the following:

Public health education: Increasing awareness among healthcare providers and the general public about the transmission of TBEV through breastfeeding is crucial. This includes educating individuals about the associated risks and preventive strategies. By promoting knowledge and understanding of TBEV transmission through breast milk, informed decision-making and appropriate counseling can be facilitated for lactating mothers. This, in turn, can help individuals make informed choices regarding breastfeeding continuation, temporary cessation, or alternative feeding options.

Improved diagnostic techniques: Developing sensitive and specific diagnostic techniques for detecting TBEV in breast milk samples is essential. These diagnostic methods should allow for the early identification of infected individuals, enabling timely interventions and appropriate management. The advancement of accurate and reliable diagnostic tools can significantly contribute to detecting and monitoring TBEV transmission through breast milk.

Individual risk assessment: Developing standardized guidelines for individual risk assessment in lactating mothers with suspected or confirmed TBEV infection is crucial. These guidelines can help healthcare providers assess the risk associated with TBEV transmission through breastfeeding on a case-by-case basis. With clear and standardized risk assessment protocols, healthcare providers can make informed decisions regarding breastfeeding continuation, temporary cessation, or alternative feeding options, taking into account the specific circumstances of each individual.

Supportive care: Providing comprehensive supportive care for infants infected with TBEV through breast milk is essential. This includes appropriate medical management, neurological monitoring, and rehabilitation services to mitigate the infection's potential complications and long-term effects. By providing specialized care tailored to the unique needs of infants affected by TBEV transmission through breast milk, healthcare providers can optimize outcomes and minimize the impact of the infection.

Collaborative research efforts: Promoting collaboration among researchers, healthcare providers, public health agencies, and policymakers is vital in advancing knowledge and addressing the challenges associated with studying TBEV transmission through breast milk. Collaborative research efforts can facilitate data sharing, exchange of best practices, and multicenter studies. By working together, researchers and stakeholders can accelerate the development of effective preventive measures and strategies to reduce the risk of TBEV transmission from mothers to infants through breastfeeding. Such collaborative initiatives can significantly impact public health policies and interventions aimed at mitigating the transmission of TBEV through breast milk.

## Conclusions

TBEV transmission through breast milk is a topic of growing interest and importance. While the overall risk of TBEV transmission through breastfeeding appears to be low, it is essential to understand the mechanisms, clinical implications, and preventive strategies associated with this transmission mode. Evidence suggests that TBEV can be present in breast milk, and there have been documented TBEV transmission from mothers to infants through breastfeeding. However, these cases are relatively rare, and the severity of infant clinical outcomes can vary. Further research is needed to address the remaining knowledge gaps, including the epidemiology, transmission mechanisms, viral load dynamics, immune response, and clinical outcomes associated with TBEV transmission through breast milk. Robust epidemiological studies, molecular investigations, and clinical trials are necessary to advance our understanding in these areas. Preventive measures play a crucial role in minimizing the risk of TBEV transmission through breastfeeding. Public health efforts should focus on tick bite prevention, vaccination programs, surveillance, and early detection of TBEV cases. Individual risk assessment, education, screening, and testing of lactating mothers can aid in informed decision-making regarding breastfeeding continuation or alternative feeding options.
